# Percutaneous removal of air-bullet gunshot: case report and literature review

**DOI:** 10.1186/s43044-020-00055-3

**Published:** 2020-05-04

**Authors:** Ali A. Alakhfash, Abdullah Alqwaee, Abdulrahman Almesned

**Affiliations:** PSCC-Qassim Prince Sultan Cardiac Center-Qassim, P O BOX 896, Buraydah, 51421 Saudi Arabia

**Keywords:** Cardiac injury, Percutaneous removal of foreign bodies, Foreign body removal in the cardiac catheterization

## Abstract

**Background:**

Cardiac air bullet injuries are rare but can be associated with significant morbidity and mortality.

**Case presentation:**

We are presenting a young male child who sustained an accidental injury to the chest by an air rifle. Bullet entered the right ventricle from the anterior part of the chest and was identified in the RV side of the interventricular septum by echocardiography and chest CT scan. There was mild pericardial effusion but no valvular injury. The bullet was removed in the cath lab, and the patient was discharged home on the second day.

**Conclusions:**

It is reasonable to try foreign body removal in the cath lab, for certain cases, and avoid cardiac surgery.

## Background

Cardiac air pellet injuries are rare but can be associated with significant morbidity and mortality. The management of such cases depends upon the degree of injury and cardiac and organ system involvement. If the cardiac involvement involves minimal injury and the pellet stuck in the heart, removal by cardiac surgery might be the option. Removal of the air pellet in the cardiac catheterization lab is an option if the interventionist feels that is feasible [1, 2].

## Case presentation

We are presenting a case of air bullet removal in the cath lab. Our case is an 11-year-old male, who sustained an air bullet gunshot while playing at home with his eldest brother in the afternoon time. Immediately after the incident, he was seen in a peripheral hospital, where primary assessment, X-ray, echocardiography and chest CT were performed. One hour later, the patient was referred to our centre for evaluation and possible cardiac surgery.

On examination: 11-year-old child, 33 kg, conscious, alert and oriented. GCS was 15/15. He was haemodynamically stable with a heart rate of 102 bpm, BP 108/68 mmHg, afebrile and respiratory rate of 24 cycle/min.

There was a small bullet entry at the anterior chest wall 2 cm above the left nipple, near to the midline. No signs of hemothorax or pneumothorax, with good bilateral air entry.

**Fig. 1:**
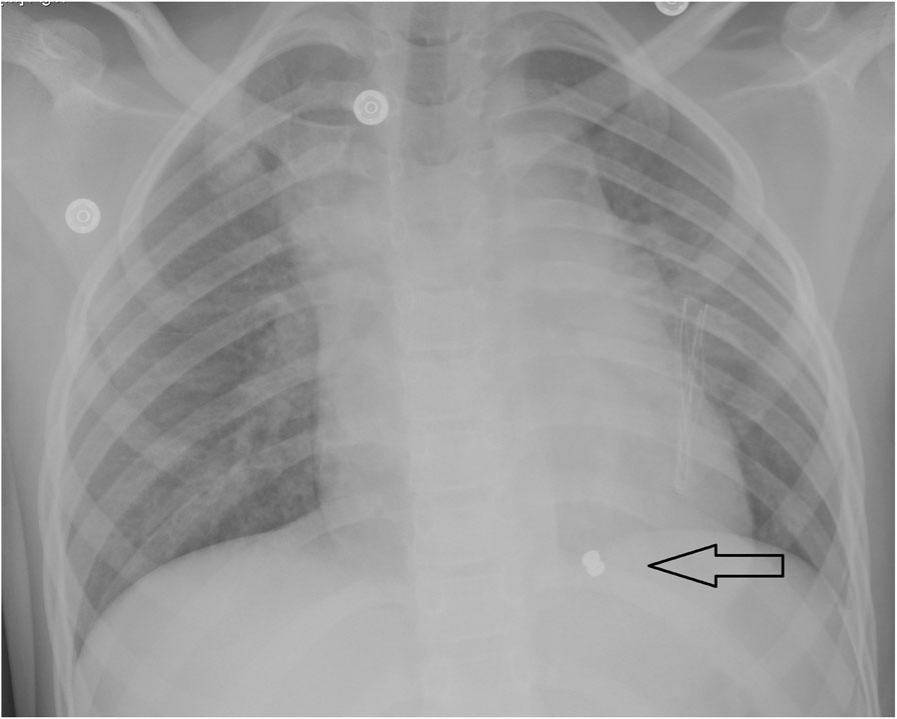
Postero-anterior chest X-ray showing a radio-opaque shadow (arrow) at the apical part of the heart more toward the right ventricle

Chest X-ray (Fig. 1) showed a radio-opaque foreign body in the left side of the sternum. Echocardiography revealed mild pericardial effusion. The foreign body (FB) was seen in the mid part of the IVS on the right ventricular side. No valvular affection and no evidence of ventricular septal defect (VSD).

**Fig. 2:**
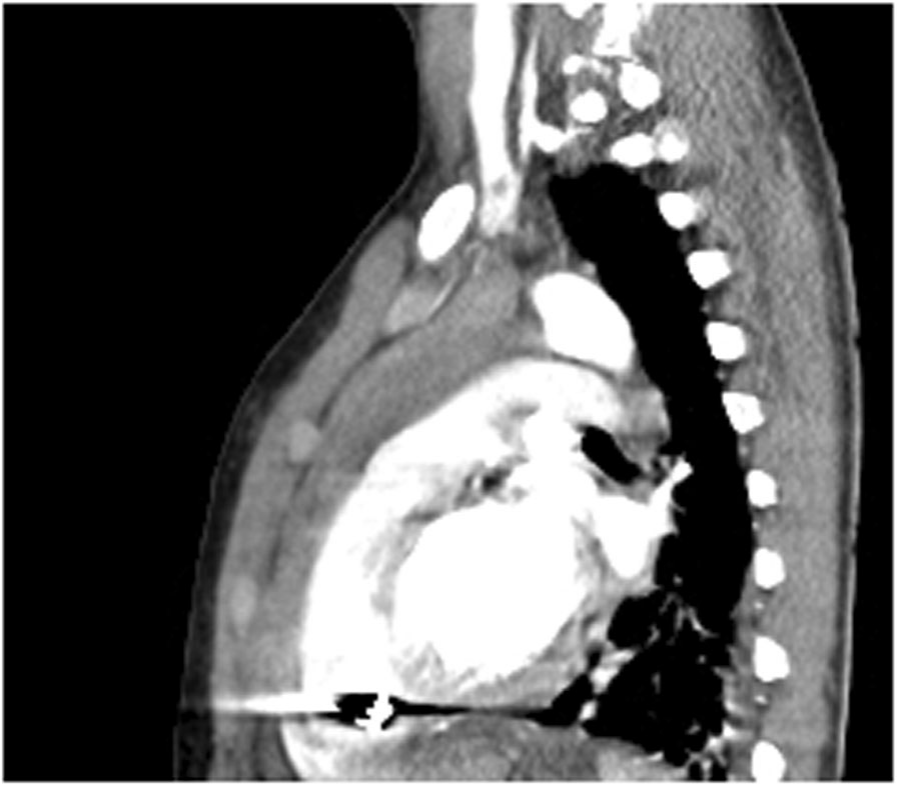
Chest CT scan with contrast, sagittal view sowing the radio-opaque pellet in the right ventricular apex with shadow artefact

A chest CT scan (Fig. 2 ) revealed a radio-opaque foreign body (bullet) seen in the projection of the base of the heart, likely within the wall of the right ventricle. There was mild pericardial effusion, with a thickness of 7 mm. No contrast extravasations during the CT scan time. There was no affection to the aorta, pulmonary artery, diaphragm, ribs, or other solid organs.

After discussion with the team and cardiac surgeon, the patient was taken to the cardiac catheterization laboratory. The family was counselled about the benefits and risks of the cardiac cath and agreed to proceed for cardiac catheterization. Surgery was not an option because the foreign body was in the RV and no valvular or septal involvement.

**Fig. 3:**
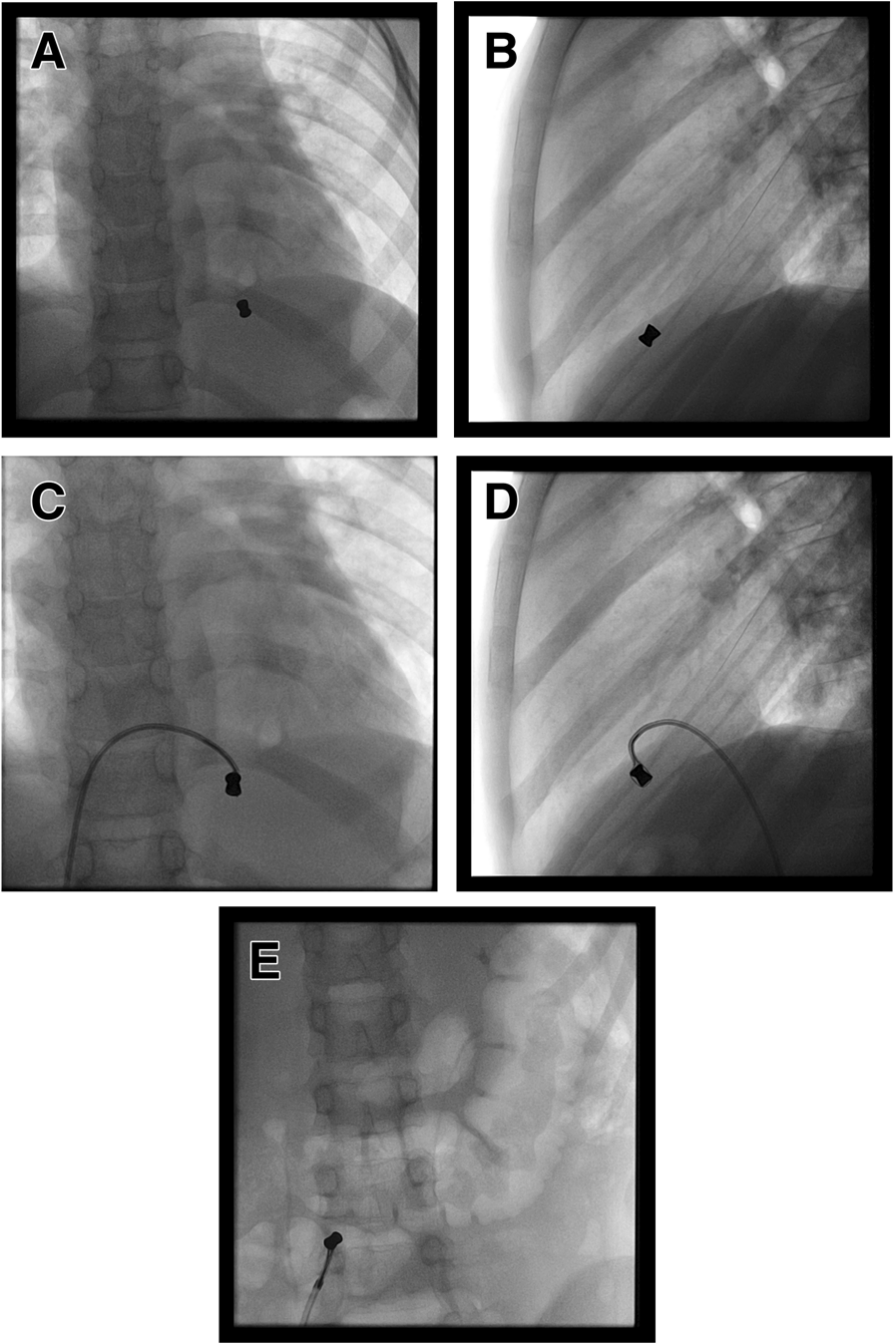
**a** One air pellet in the right ventricle (antero-posterior view). **b** Air pellet in the right ventricle lateral view. **c** The Goose Neck Snare catching the pellet (AP view). **d** Snare catching the pellet (lateral view). **e** Air pellet caught by the snare and pulled down the IVC to be exteriorized from the femoral vein

In the catheterization laboratory, under general anaesthesia, the patient was prepped and draped as protocol. The right femoral artery and vein were accessed using a 6 Fr sheath in each. Using a 5 French multipurpose catheter and then Judkins right catheter, the right side of the heart was accessed. Right ventricular hand injection performed and confirmed the position of the pellet in the RV side of the interventricular septum. The bullet was mobilized to the RV apex by the catheter. Using a 5 mm single loop, 4 Fr snare (Amplatz Goose Neck Snare), we managed to catch the bullet and exteriorize it from the heart through the femoral venous sheath (Fig. 3 and supplemental video loops).

The patient tolerated the procedure with no complications. Sheaths and catheters were removed. Haemostasis was achieved by manual pressure. Echocardiography was done after the cath and showed no tricuspid valve or right ventricular affection, with minimal pericardial effusion (as prior to cath). He was extubated in the cath lab and sent to the recovery area in stable condition.

## Discussion

Pellet (air gun), is a non-spherical projectile metallic mass designed to be shot from an air gun. Air gun pellets differ from bullet sand shots used in firearms in terms of the pressures encountered: air guns operate at pressures as low as 50 atmospheres, while firearms operate at thousands of atmospheres.

At first sight, air guns and air rifles may appear relatively harmless but they are in fact potentially lethal weapons. They use the expanding force of compressed air (or gas) to propel a projectile mass down a barrel. The projectiles are usually led to pellets or ball bearings. Technological refinements have increased the muzzle velocity and hence the penetrating power of these weapons [1, 2]. Injuries from air weapons can be serious and even fatal [3]. Air weapon injuries commonly involve teenage boys. Most are reported to occur in public places or at home. They are predominantly a result of accidental shooting by a friend, relative, or the victim himself, usually in the absence of adult supervision [4].

Several case reports have highlighted the dangers, focusing on injuries to the eye or brain [5, 6].

Approximately one-third of injuries involve the head or neck. It can result in permanent neurological injuries, including epilepsy, cognitive deficits, hydrocephalus, diplopia, visual field cut and blindness [7].

Air weapon injuries in children should be managed in the same way as any low-velocity gunshot injuries. Subcutaneous pellets are best removed. Urgent specialist referral is indicated for cranial, ocular, chest, abdominal or vascular injuries as they may require emergency surgery. Cardiac injuries may be rapidly fatal. The risk of lead intoxication from a retained air gun pellet is extremely small [8].

Retained cardiac missiles may be found free in a cardiac chamber, in the pericardial space, or partially or completely embedded in the myocardium. The initial evaluation should include chest X-ray, 12 lead ECG, 2-dimensional echocardiography and contrast-enhanced CT. The combination of two-dimensional echocardiography and contrast-enhanced CT allows accurate evaluation of the missile location, the extent of vascular and structural heart injury, and the presence and degree of pericardial effusion.

The pellet might lead to pericardial effusion and cardiac tamponade, valvular injury, intracardiac shunts, conduction defects, and/or pulmonary or systemic embolus. Patients with a missile retained in the pericardium or pericardial space may present with pericarditis. Case reports and studies have suggested that missiles completely embedded in the myocardium or pericardium/pericardial space are well tolerated and relatively safe.

The management of thoracic/cardiac pellet gun injuries should be based on the presentation and stability of the patient and the location of the retained pellet. Expectant, non-operative management may be considered for the stable, asymptomatic patient with intramyocardial or pericardial pellets or bullets. In contrast, intracavitary missiles, missiles with valvular injuries or those partially embedded in the myocardium should be removed to prevent embolization or thrombosis. A low threshold for surgical intervention should be used in symptomatic patients. Careful observation and imaging must contribute to the management decisions for any patient presenting with a cardiac missile. Pericardial effusion might need pericardiocentesis and/or cardiac surgery to prevent cardiac tamponade [9, 10].

There are reports of patients, who required surgical intervention for penetrating injuries to the heart, including window pericardiotomy for hemopericardium, exclusion of the cardiac apex for a traumatic ventricular septal defect, and RV injury leading to pericardial effusion and cardiac tamponade requiring pericardial window [11, 12].

Most of the reported injuries to the heart or chest were treated surgically with surgical removal of the pellet [13].

There is a case report of a 15-year-old boy who sustained air pellet injury to the heart leading to pericardial effusion and tamponade. The bullet was in the right ventricle. The patient was taken to the OR and evacuation of pericardial tamponade was done. After the tamponade was relieved, a trans-oesophageal echocardiogram was performed to locate the bullet, which could not be found in the ventricle. Chest and abdominal radiography confirmed that the bullet had migrated retrogradely down into the inferior vena cava. The chest was closed and the patient was transferred from the operating theatre to the interventional radiology department. Under fluoroscopy, the bullet was pulled down into the right common femoral vein and extracted by venorrhaphy [14].

If the pellet is in the left ventricle, there is a potential for embolization and the pellet should be removed, most probably through surgical exploration [15].

In our case, the patient was taken directly to the cath lab and the pellet was snared successfully with no need for surgical exploration or venorrhaphy. We did not find a report of similar intervention for such cases.

In Saudi Arabia, civilian possession of guns is regulated by law. Most conventional air weapons in the KSA require a license, and children under 14 years are not allowed to use an air weapon if not supervised by a person aged 21 years or more [https://www.gunpolicy.org/firearms/region/saudi-arabia].

## Limitations

This is a case report. Management of thoracic/cardiac pellet gun injuries should be based on the presentation and stability of the patient; the location of the retained pellet and the expertise of the medical team.

## Conclusion

Air weapons are capable of inflicting serious and potentially fatal injury in children. The management of thoracic/cardiac pellet gun injuries should be based on the presentation and stability of the patient and the location of the retained pellet. Removal of the pellet in the cath lab is a possibility if the pellet is in an accessible area of the heart or vascular system.

## Supplementary information

**Additional file 1.**

## Data Availability

Cath lab data and loops are available for anyone interested.

## Abbreviations

BP: Blood pressure
FB: Foreign body
Fr: French
GCS: Glasgow Coma Scale
KSA: Kingdom of Saudi Arabia
RV: Right ventricle
VSD: Ventricular septal defect

## References

[1] Jin Y,Haitao L,Cheng W,Wang X,Han R,Li R, et al. The experimental and numerical investigation on the ballistic limit of BB-Gun pellet versus skin simulant.Forensic Sci Int. 2019;298:393-397. doi: 10.1016/j.forsciint.2019.02.033. Epub 2019 Mar 15. 10.1016/j.forsciint.2019.02.033 30947143

[2] Aslan S, Uzkeser M, Katirci Y, Cakir Z, Bilir O, Bilge F (2006). Air guns: toys or weapons?. Am J Forensic Med Pathol.

[3] Charlier P, Alvarez JC, Durigon M, de la Grandmaison GL (2012). Unusual death by rubber bullet: should these guns be reclassified as lethal weapons?. Am J Forensic Med Pathol..

[4] Ceylan H, McGowan A, Stringer MD (2002). Air weapon injuries: a serious and persistent problem. Arch Dis Childhood.

[5] Giran G,Bertin H,Koudougou C,Sury F,Croisé B,Laure B. About a pediatric facial trauma. J Stomatol Oral Maxillofac Surg. 2019;120(2):154-156. doi: 10.1016/j.jormas.2018.11.001. Epub 2018 Nov 12. 10.1016/j.jormas.2018.11.001 30439549

[6] Saunte JP, Saunte ME (2006). 33 cases of airsoft gun pellet ocular injuries in Copenhagen, Denmark, 1998-2002. Acta Ophthalmol Scand..

[7] Kumar R, Kumar R, Mallory GW, Jacob JT, Daniels DJ, Wetjen NM (2016). Penetrating head injuries in children due to BB and pellet guns: a poorly recognized public health risk. J Neurosurg Pediatr.

[8] Keller JE, Hindman JW, Kidd JN, Jackson RJ, Smith SD, Wagner CW (2004). Air-gun injuries: initial evaluation and resultant morbidity. Am Surg.

[9] Klein JA, Nowak JE, Sutherell JS, Wheeler DS (2010). Nonsurgical management of cardiac missiles. Pediatr Emerg Care..

[10] Gandhi SK, Marts BC, Mistry BM, Brown JW, Durham RM, Mazuski JE (1996). Selective management of embolized intracardiac missiles. Ann Thorac Surg.

[11] DeCou JM, Abrams RS, Miller RS, Touloukian RJ, Gauderer MW (2000). Life-threatening air rifle injuries to the heart in three boys. J Pediatr Surg..

[12] Wascher RA, Gwinn BC (1995). Air rifle pellet injury to the heart with retrograde caval migration. J Trauma..

[13] Nakamura DS, McNamara JJ, Sanderson L, Harada R (1983). Thoracic air gun injuries in children. Am J Surg..

[14] McLaughlin RL, Analitis S, Van Vleet S, Pederson R (2008). Right ventricular gunshot wound with retrograde embolization. J Trauma Nurs.

[15] Greenlees G, Govewalla P, Haqzad Y, Sharkey A, Cartwright N. Penetration of the heart by an air gun pellet. A case without significant effusion or valvular injury. Ann Thorac Surg. 2018 Dec 17. 10.1016/j.athoracsur.2018.11.034 30571952

